# Intercostal artery pseudoaneurysm following thoracentesis: multi-modal imaging and treatment

**DOI:** 10.1186/s12880-019-0333-5

**Published:** 2019-04-27

**Authors:** Kaitlyn P. Casper, Paul J. Sanchirico, David C. Pfeiffer

**Affiliations:** 1 WWAMI Medical Education Program, University of Washington School of Medicine, 1959 NE Pacific St, Seattle, WA 98195 USA; 2 St Joseph Regional Medical Center, 415 6th St, Lewiston, ID 83501 USA; 3 WWAMI Medical Education Program and Department of Biological Sciences, University of Idaho, 875 Perimeter Drive, Moscow, ID 83844-3051 USA

**Keywords:** Pseudoaneurysm, Thoracentesis, Intercostal artery, Hemothorax, Coil embolism, Multi-modal imaging

## Abstract

**Background:**

A pseudoaneurysm occurs as the result of a contained rupture of an arterial wall, yielding a perfused sac that communicates with the arterial lumen. Pseudoaneurysm of an intercostal artery is an extremely rare event but it carries with it a significant risk of rupture and subsequent hemothorax. It must be considered as a potential complication of thoracentesis.

**Case presentation:**

Here, we report a rare case of an intercostal artery pseudoaneurysm following thoracentesis in an 82-year old male. The patient presented with respiratory distress 1 day after a therapeutic thoracentesis had been performed. Computed tomography (CT) with contrast revealed a left intercostal pseudoaneurysm with hemothorax and adjacent compressive atelectasis. Doppler ultrasound revealed bidirectional blood flow in the pseudoaneurysm sac. An intercostal arteriogram and thoracic aortogram aided in confirmation of the pseudoaneurysm and successful treatment with coil embolization.

**Conclusions:**

An intercostal pseudoaneurysm complication following thoracentesis is very rare but important to rule out as a possible cause of hemothorax after the procedure. Capturing this finding with the aid of multiple imaging modalities allowed for diagnostic certainty and rapid treatment with coil embolization, leading to a successful patient recovery.

## Background

Thoracentesis is a commonly performed procedure in the diagnosis and possible treatment of pleural effusion. Typical complications of the procedure include pneumothorax, bleeding including hemothorax, and re-expansion pulmonary edema [1]. A pseudoaneurysm results from damage to an arterial wall in a manner that permits blood to dissect into tissues of the vessel wall, forming a perfused sac that communicates with the arterial lumen [2, 3]. Pseudoaneurysm of an intercostal artery is an extremely rare condition, with relatively few documented cases in the literature. Previous reports have documented it as a complication of chest trauma or surgical procedures [4–13]. It carries with it a significant risk of rupture and subsequent hemothorax and therefore prompt and accurate identification is important. We describe a case of an intercostal pseudoaneurysm complication of a thoracentesis that was documented on multiple imaging modalities and successfully treated with coil embolization.

## Case presentation

**Fig. 1:**
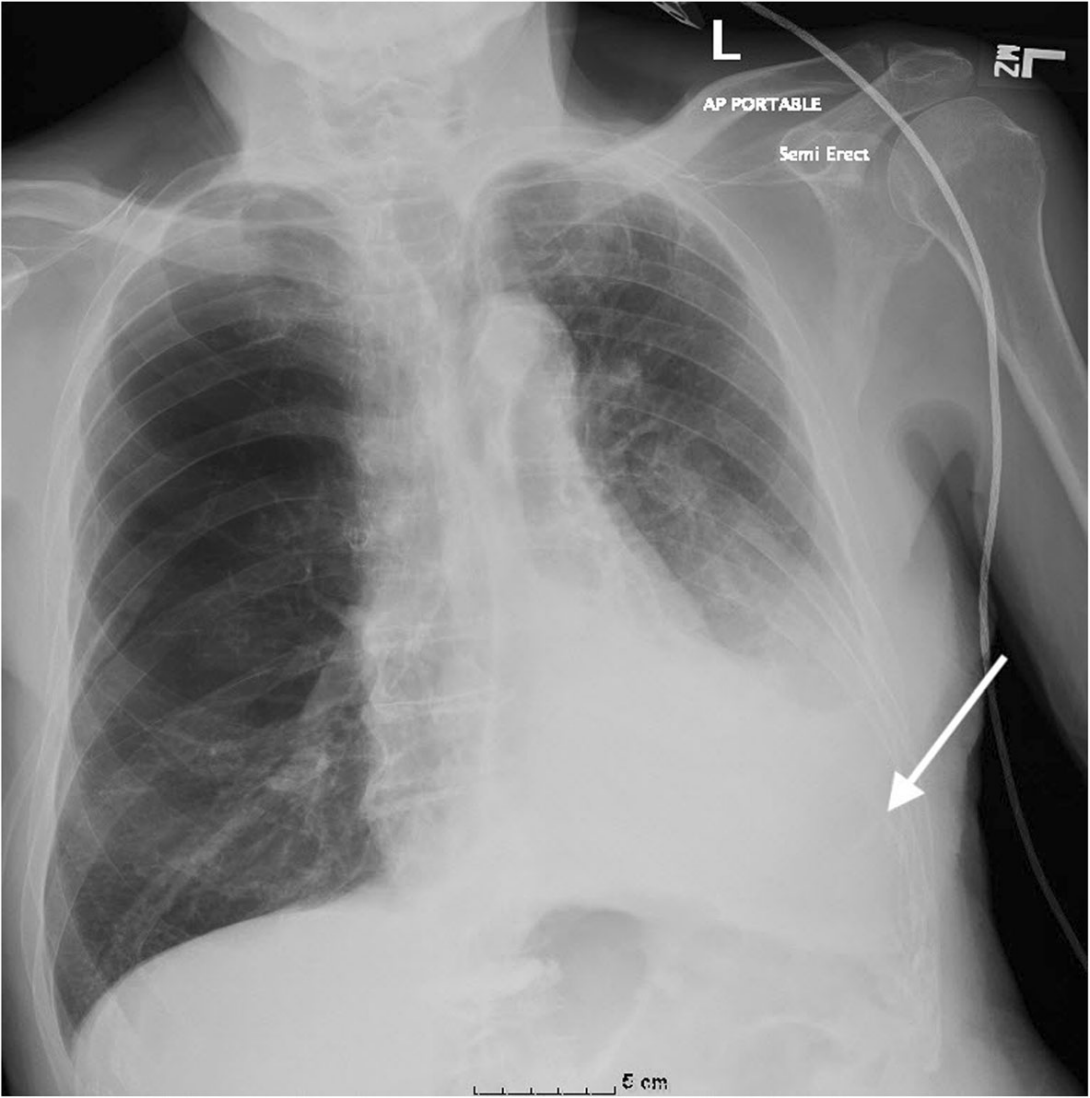
AP (anteroposterior) chest plain radiograph in an 82-year old male, following thoracentesis. Note the significant pleural effusion with compressive atelectasis (arrow) in the left lower hemithorax

**Fig. 2:**
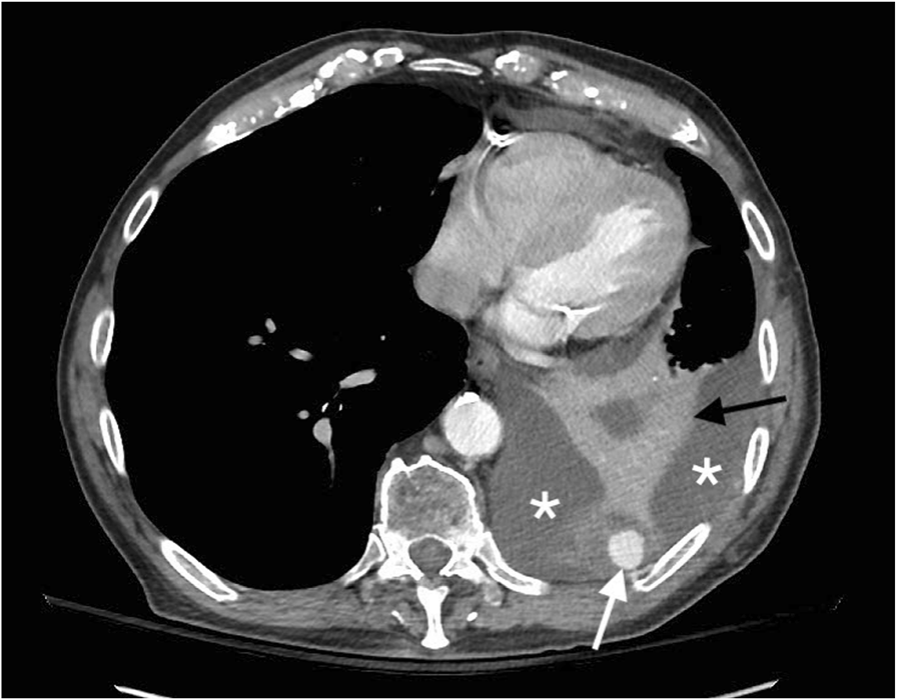
IV contrast-enhanced computed tomography (CT) image, axial projection. The left intercostal artery pseudoaneurysm (white arrow) with hemothorax (asterisks) and adjacent compression atelectasis (black arrow) can be seen

**Fig. 3:**
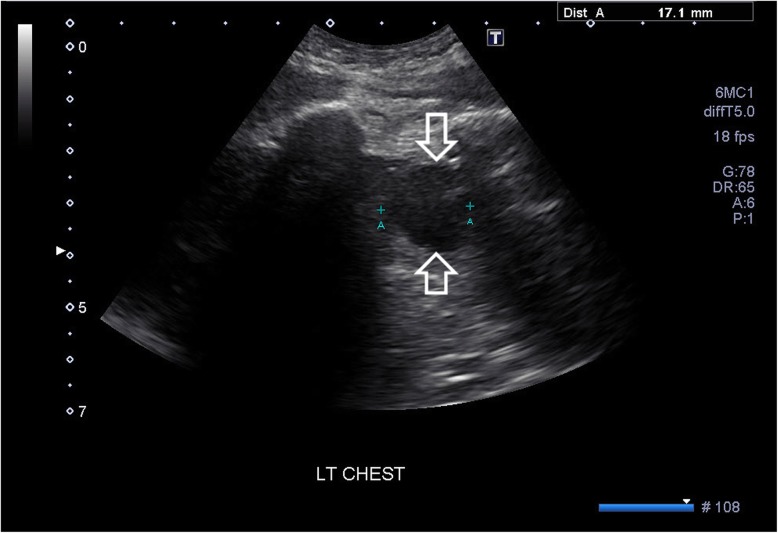
Grey scale ultrasound image of the left intercostal artery pseudoaneurysm. Arrows delineate the pseudoaneurysm

**Fig. 4:**
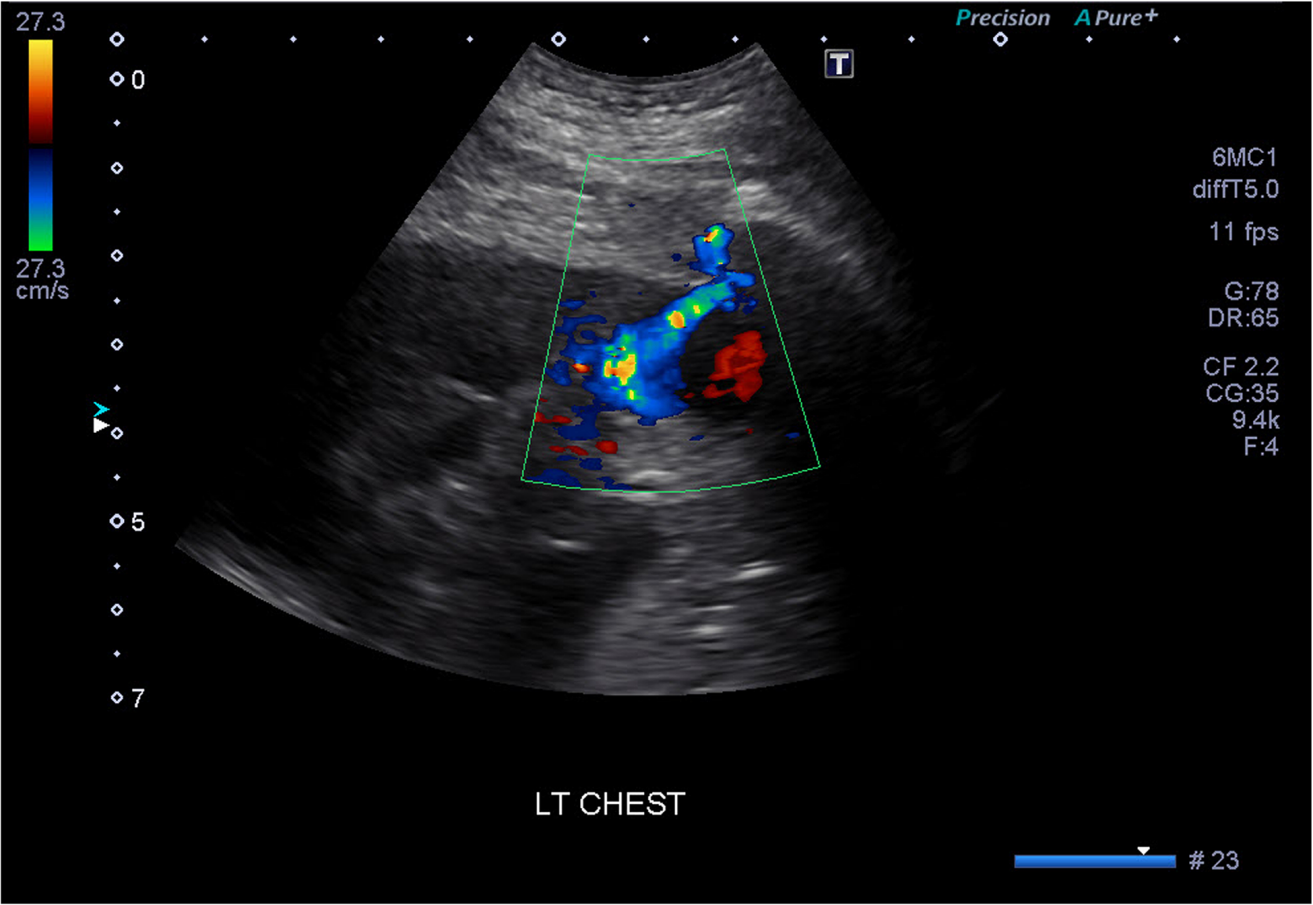
Color Doppler ultrasound image of the intercostal artery pseudoaneurysm. Note the characteristic yin-yang (red-blue) blood flow present in the pseudoaneurysm

An 82-year-old male was admitted to the emergency department for worsening shortness of breath and hypoxia. He was admitted a week after he was diagnosed with a left ninth rib fracture secondary to a fall. He had long-standing history of chronic obstructive pulmonary disease, coronary artery disease, and peripheral vascular disease. Chest radiographs revealed a left pleural effusion and possible infiltrate. The patient was initially treated with a nebulizer, prednisone, and empiric antibiotic coverage with ceftriaxone and azithromycin. The patient failed to improve with the medical interventions and a therapeutic thoracentesis was performed. The thoracentesis was completed with ultrasound guidance, with the puncture made above the 11th rib at mid chest on the left. The pleural effusion was found to be frank blood. No immediate complications were noted, and the patient was taken to recovery. The next day the patient was found to be in respiratory distress. A chest x-ray revealed an opaque left hemithorax that was likely rapid accumulation of pleural fluid (Fig. 1). A follow-up contrast-enhanced computed tomography (CT) of the chest performed during the arterial phase revealed a left intercostal pseudoaneurysm with hemothorax and adjacent compressive atelectasis (Fig. 2). Ultrasound of the left chest wall was performed (Fig. 3) directly over the thoracentesis site and doppler flow revealed bidirectional fluid flow, indicating the presence of a large pseudoaneurysm (Fig. 4).

**Fig. 5:**
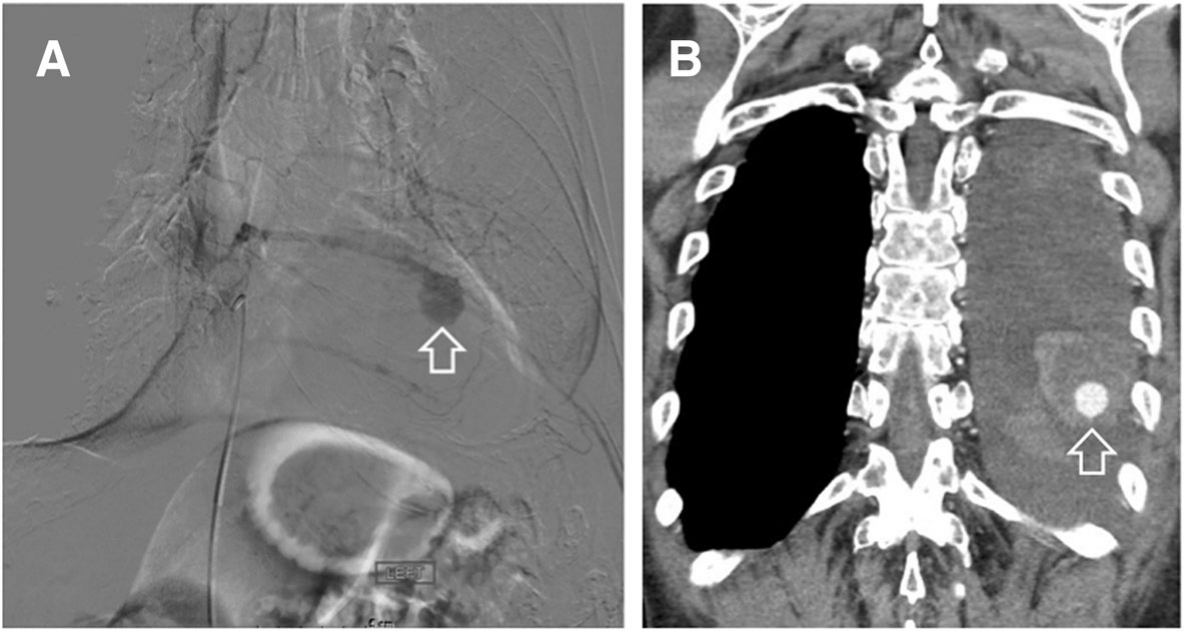
**a** Selective digital subtraction angiogram of the intercostal artery pseudoaneurysm (arrow). **b** IV contrast-enhanced computed tomography (CT) image, coronal projection, depicting the pseudoaneurysm (arrow)

**Fig. 6:**
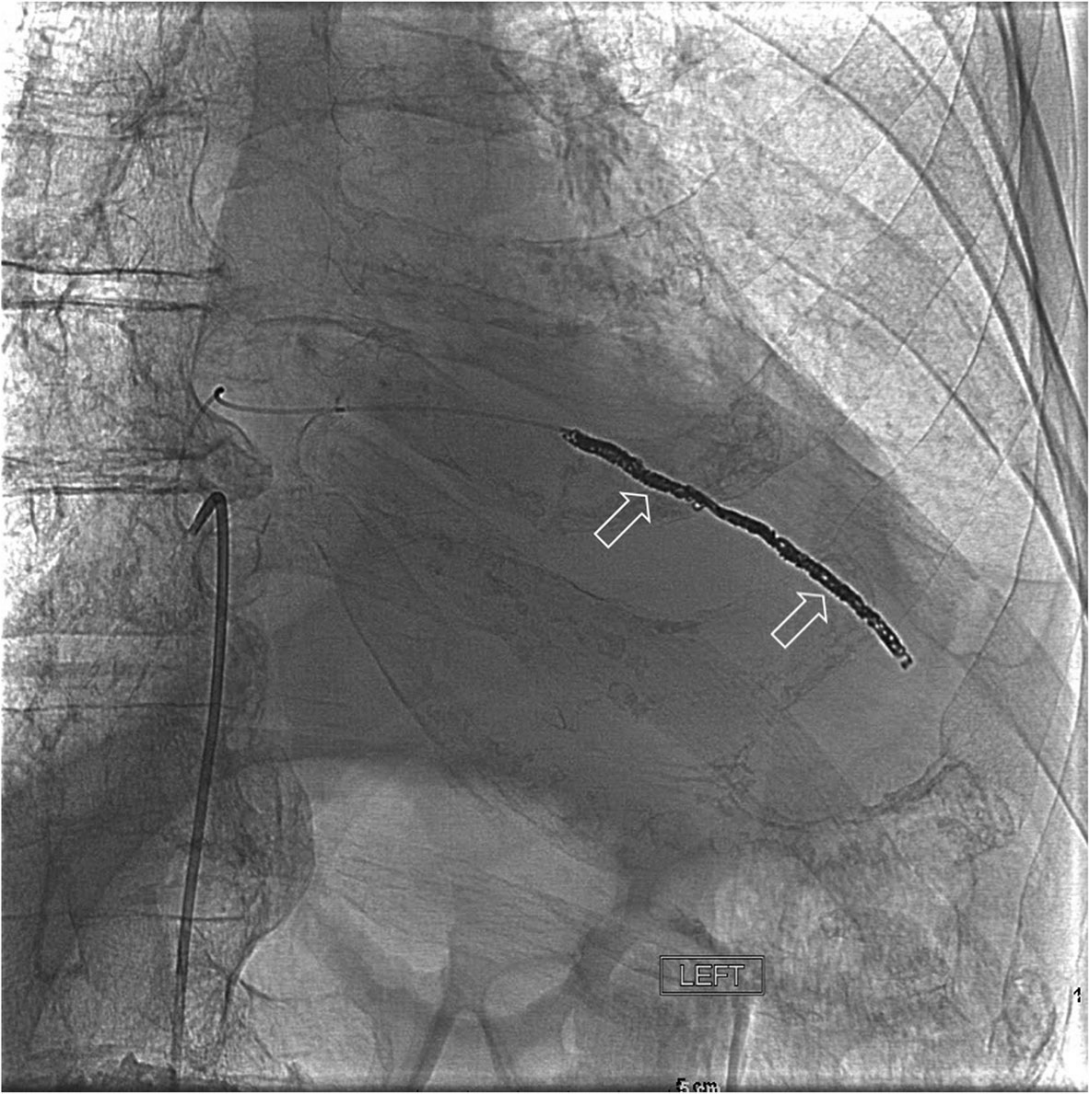
Digital subtraction angiogram image demonstrating successful occlusion of the intercostal artery pseudoaneurysm by means of coil embolization. The coils are clearly visible (arrows)

Following identification of the left intercostal pseudoaneurysm, the patient underwent a thoracic aortogram and multiple-level left intercostal angiogram (Fig. 5) under IV conscious sedation. Selective catheterization of the T5, T6, and T7 intercostal arteries was unsuccessful in identifying the pseudoaneurysm. Selective catheterization of T10 and T11 intercostal arteries was performed with a C2 Cobra catheter, following multiple catheter exchanges due to the patient’s atherosclerotic vessels. The pseudoaneurysm was ultimately found to have a left T10 origin and the C2 Cobra catheter was exchanged for a microcatheter. Once access was gained, coil embolization of the pseudoaneurysm was performed with a series of 15 Axium micro coils. Significant room was left on both sides of the pseudoaneurysm and a follow-up angiogram was performed via the microcatheter, then a 5-French Cobra catheter. The follow-up angiogram demonstrated no further filling of the pseudoaneurysm (Fig. 6). The catheter was removed and a Perclose device was placed in the left groin for hemostasis. Following completion of the procedure, the patient was taken to recovery. The patient proceeded to return to his baseline following medical management during the remainder of his hospital stay and was discharged home after 5 days.

## Discussion

Thoracentesis is a very common procedure used to remove pleural fluid for diagnostic or therapeutic purposes. The most common complications of the procedure include pneumothorax, bleeding includinghemothorax, and re-expansion pulmonary edema [1]. Following the introduction of ultrasound guiding thoracentesis, the complication rates have significantly decreased due to improved accuracy of needle placement [14]. As in our case, hemothorax is to be considered if the patient has a rapid accumulation of pleural fluid or signs of respiratory distress.

An intercostal pseudoaneurysm is an extremely rare phenomenon, seldomly described in literature. Etiology of previously described intercostal artery pseudoaneurysms includes blunt thoracic trauma [7, 8, 12], and surgical procedures including thoracoscopic sympathectomy [4], percutaneous biliary procedure [5], laparoscopic procedure [6], sternotomy [9], aortic valve implantation [11], and biopsy [10, 13]. To the best of our knowledge, only one other case reported this complication as the result of thoracentesis; however, in that case the patient was unable to undergo successful embolization of the pseudoaneurysm [15].

Our patient’s intercostal pseudoaneurysm was captured with the aid of three different imaging modalities prior to treatment intervention. The pseudoaneurysm was initially detected on the contrast-enhanced CT. The procedure was performed during arterial phase and this played a pivotal role in the diagnostic process. Doppler ultrasound identifying the possible pseudoaneurysm revealed a “yin-yang sign” which can be characteristic of the complication. This radiological sign has been described to represent the bidirectional flow of blood in the pseudoaneurysm sac and is helpful in the diagnostic confirmation [8, 13].

The gold standard for diagnosing pseudoaneurysms on imaging is conventional angiography, however, less invasive modalities are increasing in use including ultrasonography, computed tomographic angiography (CTA), and magnetic resonance angiography (MRA) [8]. CTA and MRA both have been demonstrated to be reliable, high-quality imaging tools for the evaluation of vascular abnormalities in general and provide the advantages of rapid examination and short scanning times [17–19]. Disadvantages associated with CTA include patient exposure to ionizing radiation [20, 21]. Contrast-enhanced MRA carries with it the risk of adverse reactions to gadolinium-based contrast material, a serious concern in patients with poor renal function. CT techniques that use less contrast agent could provide viable alternatives, although more studies on their effectiveness in vascular imaging are needed [19]. Newer contrast-enhanced MRA methods that utilize dose reduction at 3.0 T, novel contrast agents, parallel imaging methods, and time-resolved imaging for contrast kinetics have been shown to be effective in the evaluation of other vascular disorders [22–24] and may have potential in the diagnosis of pseudoaneurysms.

Early treatment of the pseudoaneurysm before rupture is critical in the success of the patient’s recovery. If rupture did occur, the patient would be at risk of a possible life-threatening hemorrhage [8]. Currently, coil embolization is the most common treatment of intercostal artery pseudoaneurysms, followed by covered stent placement [8, 16]. In previously reported cases, the return of blood flow has been documented following embolization. These cases required re-treatment with thrombin, *n*-butyl cyanoacrylate or re-embolization at a different location [8, 16]. Our patient had successful primary embolization with Axium coils and a follow up angiogram revealed no further filling of the aneurysm.

## Conclusion

An intercostal artery pseudoaneurysm as a complication of thoracentesis is an extremely unusual condition but it is important to rule out as a possible cause of hemothorax after the procedure. Capturing this finding with the aid of multiple imaging modalities allowed for diagnostic certainty and rapid treatment with coil embolization, leading to a successful patient recovery.

## Abbreviation

CT: computed tomography
